# MOF-5 derived carbon as material for CO_2_ absorption

**DOI:** 10.1039/c9ra01786k

**Published:** 2019-06-12

**Authors:** Wojciech Kukulka, Krzysztof Cendrowski, Beata Michalkiewicz, Ewa Mijowska

**Affiliations:** Nanomaterials Physicochemistry Department, West Pomeranian University of Technology, Szczecin Piastów Av. 45 Szczecin 70-311 Poland wojciech_kukulka@zut.edu.pl emijowska@zut.edu.pl; Institute of Chemical and Environment Engineering, West Pomeranian University of Technology, Szczecin Pulaskiego St. 10 Szczecin 70-322 Poland

## Abstract

In our study we prepared MOF-5 derived carbon to reveal the thermodynamics of CO_2_ absorption processes in great detail. Porous carbon material was prepared from a metal–organic framework (MOF-5) *via* carbonization at 1000 °C. The obtained structure consists only of carbon and exhibits a BET specific surface area, total pore volume and micropore volume of 1884 m^2^ g^−1^, 1.84 cm^3^ g^−1^ and 0.59 cm^3^ g^−1^, respectively. Structural analysis allowed the assumption that this material is an ideal candidate for efficient CO_2_ absorption. The CO_2_ uptake was 2.43 mmol g^−1^ at 25 °C and 1 bar. Additionally, the absorption over a wide range of temperatures (25, 40, 60, 80 and 100 °C) and pressures (in range of 0–40 bar) was investigated. It is shown that the CO_2_ absorption isotherm fits a multitemperature Sips model. The calculated Sips equation parameters allows the isosteric heat of adsorption to be obtained. The isosteric heat of adsorption for CO_2_ decreased substantially with an increase in surface coverage by gas molecules. This indicates a negligible intermolecular interaction between CO_2_ molecules. A decrease in the isosteric heat of adsorption with surface coverage is a result of the disappearance of favourable adsorption sites.

## Abstract 2

In our study we prepared MOF-5 derived carbon to reveal the thermodynamics of CO_2_ absorption processes in great detail.

## Graphical abstract

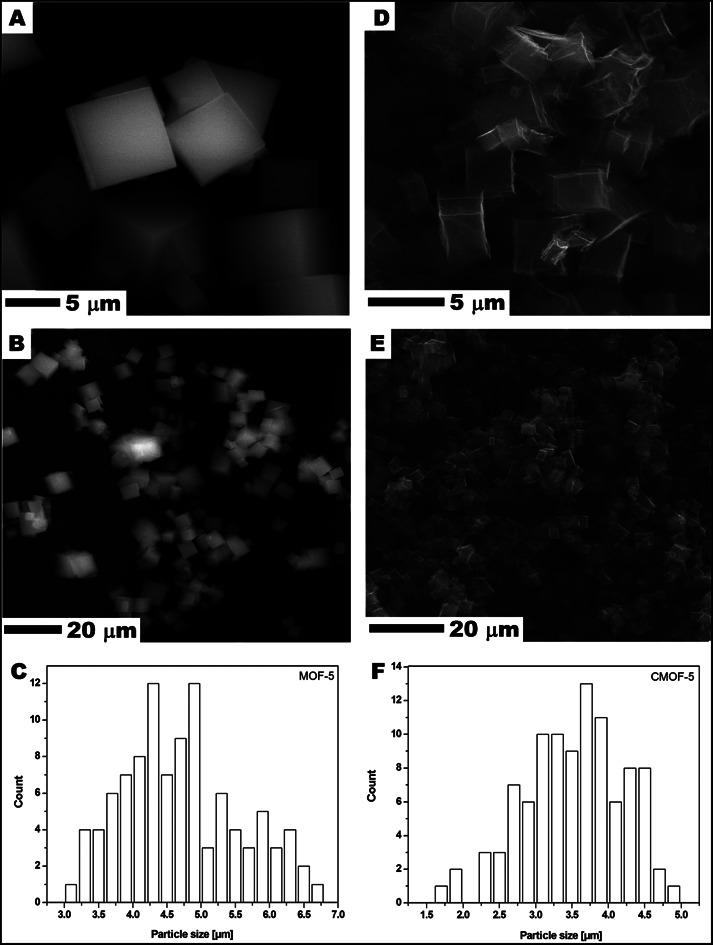

## 1. Introduction

The 21st century poses huge challenges for all scientists, in particular those involved in energy storage and environmental protection. Many different questions can be answered by studies on the absorption of various gases. The most commonly studied gases for potential applications are hydrogen (H_2_), carbon dioxide (CO_2_) and methane (CH_4_). The production and use of hydrogen as a source of energy is one of the development priorities. It is the basis for the development of a new industry of clean energy technologies. In the future, hydrogen will be used in fuel cells that are part of large energy systems. However, to do this, the two most important hydrogen problems must be solved – storage and transport.^1^ Another gas, which is considered in terms of an alternative source of energy and reducing pollution of the natural environment, is methane. It could successfully replace gasoline and diesel fuel in vehicles. Its advantages also include natural abundance and clean combustion. However, as in the case of hydrogen, the biggest obstacle to its widespread use is its storage. One of the most serious threats about the natural environment is global warming resulting from the greenhouse effect caused by excessive CO_2_ emission from different sources^2^ like the steel or automobile industries. Carbon dioxide traps radiation, creating ground-level ozone which leads to disturbances in the daily amplitude of the air temperature. It warms up oceanic waters, thereby reducing their ability to adsorb CO_2_ from the atmosphere, creating a vicious circle. Increased temperature also causes melting of glaciers and continuous increase of the water level. It certainly has an impact on climate change. Carbon dioxide emission certainly also has an impact on human health and well-being. Thus, it is necessary to limit its emission by using specialized filters and absorbers. Therefore, we need more and more novel materials with high gas absorption capacity for all abovementioned gases and we wish that this adsorption takes place effectively at room temperature.

Many different types of materials have already been tested in this regard. Zeolites^3–8^ and porous carbon materials^9–14^ were of particular interest. Recently, another group of potential candidates for absorption of gases has appeared – metal–organic frameworks. They have been examined for the absorption of the abovementioned gases.^15,16^ The first reports on the absorption of gases in MOFs was published already in the middle of the last decade.^17–19^ Since then, various MOF structures have been investigated for gas absorption. Until now, the absorption properties have been tested for such metal–organic materials as MOF-5,^20–22^ MOF-177,^23–25^ ZIF-8,^26,27^ IRMOF,^28,29^ HKUST,^30,31^ MIL-100/101 (ref. 31–34) and UiO-66/Zr-MOF.^35,36^ The most interesting property of metal–organic structures is the ease to obtain highly porous carbon materials after simple carbonization. This enables a number of new materials to be tested for various applications, including gas absorption. Metal–organic structures are often unstable and prone to damage, for example as a result of prevailing humidity in the air. So, carbonization is a very simple solution to obtain a stable material under various conditions. The carbonization of MOF also can generate highly porous carbon products and hence the high specific surface area and total pore volume.^37^ The optimization of the carbonization parameters of metal–organic structures is widely studied in the literature.^38^ The first absorption measurements of hydrogen, methane and carbon dioxide in the carbonized metal–organic structures were also performed. The absorption measurements of different gases for carbonized MOF-1,^39^ MOF-5,^40^ MIL^41–43^ and ZIF^44–49^ structures were performed. The detailed data are presented in Table 4.

Carbonized MOF structure shows the advantage over other carbon structures, like amorphous carbon, with the uniform structure and control over the MOF structures properties with the synthesis parameters. Recent articles showed that with control of the synthesis and carbonization parameters, different properties can be tuned depending on the needs.^38,50^ The MOF-5 structure are not without disadvantages. The main disadvantages are the stability of the MOF-5 in the presence of even trace amounts of moisture and the compounds toxicity during synthesis. Both of this problems can be eliminated by carbonization of the MOF-5 structure directly after synthesis. The MOF-5 structure shows high surface but after exposition to the trace amounts of moisture, their crystal structure starts to decomposed.^51^ In results of that specific surface area of MOF-5 structures drops radically. The thermal transformation of MOF-5 to the MOF-5 derived carbon structures allows to maintain the pristine crystals size and shape with similar surface area. After carbonization obtained MOF-5 derived carbon structures are immune to water and shows stability at higher temperature.^52,53^ Second disadvantage was the high toxicity of DMF used for the synthesis of MOF-5 and due to this high cost of the material production. Our recent publication showed that DMF used for MOF-5 synthesis, after separation from obtained structure and by-products can be reused.^54^ Additionally, recent presented data show recovering and synthesis MOF structures from terephthalic acid from polyethylene terephthalate (PET) waste like used plastic bottles.^54^ Since MOF-5 can be synthesized from DMF and PET waste the MOF productivity problem can be resolved.

Detailed research on the mechanism of MOF-5 carbonization, were previously reported elsewhere.^38,55,56^ In our previous research, TGA analysis was performed in the temperature range from 25 to 1000 °C under inert gas flow (argon).^38^ The observed weight loss were assigned to the: removal of water and residual solvent molecules (in the range from 25 to 200 °C); thermal decomposition of organic ligand molecules and the formation of CO_2_ and benzene after the breaking of carboxylic bridges between benzene rings and Zn_4_O clusters (in the range from 400 to 550 °C),^57^ carboreduction of ZnO, in which carbonaceous materials deoxidize ZnO and later evaporate forming mainly CO_2_ and CO. This process starts at about 750 °C and intensify with the temperature.^38^ The effect of zinc oxide nanostructure formation (spherical and rod-like structures), growth and thermal extraction induces cracking and cavity formation in the carbonized structure. Such structural distortions may reduce the surface area of the whole microporous structure, causing a deterioration of the physical properties of the carbonized MOF-5. Cracking and the appearance of voids take place regardless of the shape of zinc oxide nanostructures; however; the concentration of defects in the carbonized MOF-5 increases with nanorod formation.^38^ This negative effect can be minimalize by long thermal carbonization at high temperatures.^38,58^

In this work, we focus on the absorption properties of carbon dioxide by the carbonized MOF-5 structure. The thermodynamics of the absorption process in our material has also been investigated in great details. Therefore, we could fill the gap in current state of the art – there is a lack of report on it.

## 2. Experimental

### 2.1 Materials

Hydrochloric acid (36%) and *N*,*N*-dimethylformamide were purchased from Chempur (Poland). Zinc nitrate hexahydrate (Zn(NO_3_)_2_·6H_2_O) and terephthalic acid (C_6_H_4_(COOH)_2_) were bought from Sigma Aldrich.

### 2.2 Preparation of the carbonized MOF-5

The MOF-5 was prepared according to literature.^55^ Briefly, 1.65 mmol of zinc nitrate hexahydrate and 0.89 mmol terephthalic acid were dissolved in 2.34 mol *N*,*N*-dimethylformamide (DMF). The mixture was sonicated to obtain homogenous solution, transferred into the autoclave and stirred for 48 hours at the temperature of 150 °C. The obtained white powder was vacuum dried at 110 °C in order to remove the solvent. Further, ceramic boat containing MOF-5 cubic crystals was inserted into the tubular furnace. The sample was heated under Ar flow to 1000 °C for 2 hours. After carbonization, derived carbon sample was immersed in 36% hydrochloric acid for 48 hours. Finally, carbonized MOF-5 was filtrated, washed with distilled water and ethanol, and dried in air.

### 2.3 Characterization techniques

The morphology and chemical composition of the samples was analysed with the scanning electron microscopy (SEM, VEGA3 TESCAN) and transmission electron microscope (Fei Tecnai G2 F20 S Twin with energy dispersive X-ray spectroscopy). Specific surface area analysed through adsorption using the Brunauer, Emmett and Teller (BET) isotherm was performed with a Quadrasorb SI (Quantachrome Instruments). X-ray diffraction (XRD) patterns were carried out using X'Pert Philips Diffractometer with Cu lamp (Kα1 = 1.54056 Å) to investigate the crystal composition of the samples. Thermogravimetric analysis (TGA) was carried out on 10 mg samples using the DTA-Q600 SDT TA Instrument at the heating rate of 5 °C min^−1^ from room temperature to 1000 °C in air. Raman spectra were performed using *via* Raman Microscope (Renishaw) with the excitation wavelength of 785 nm. The adsorption capacities of carbon dioxide were measured using a Sievert-type volumetric apparatus (IMI, Hiden Isochema, U.K.).

## 3. Results and discussion

**Fig. 1:**
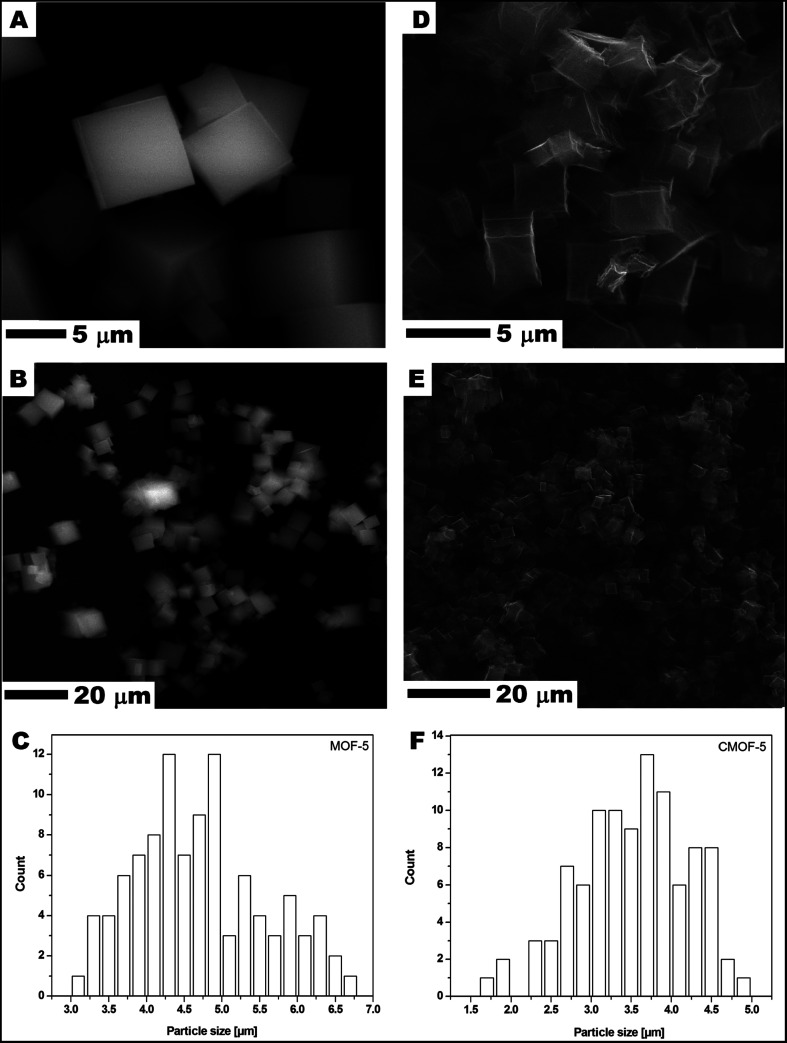
SEM images of MOF-5 (A and B) and carbonized MOF-5 (D and E) and particle size distribution of MOF-5 (C) and carbonized MOF-5 (F).

**Fig. 2:**
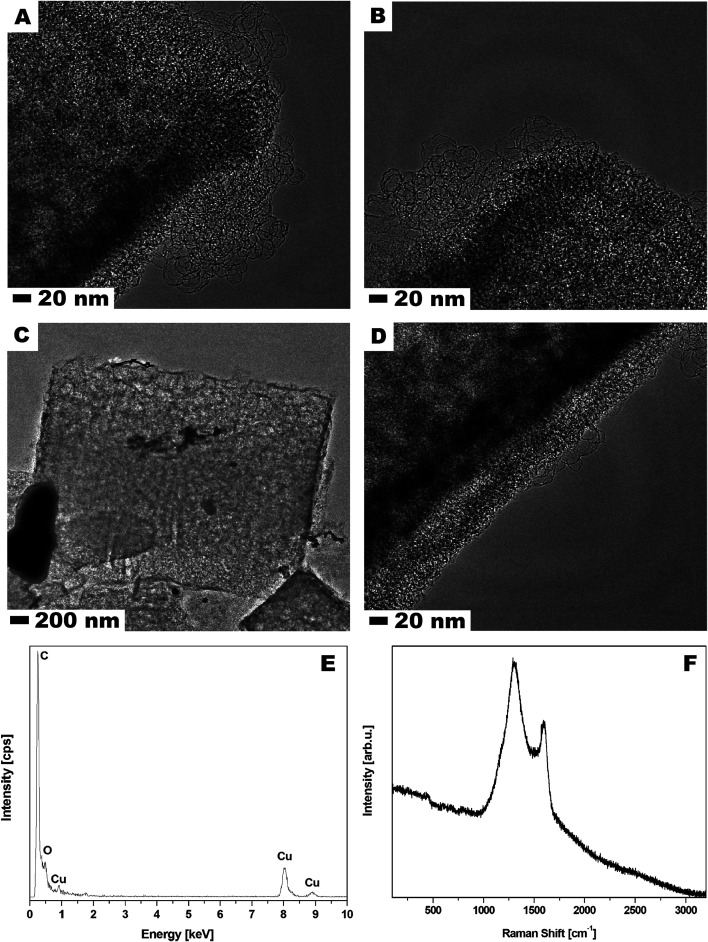
TEM images (A–D), EDS analysis (E) and Raman spectra (F) of the carbonized MOF-5.

Scanning electron microscopy (SEM) was used to examine MOF-5 and carbonized MOF-5 morphology – size, shape and surface structure (Fig. 1). The MOF-5 presents cube-shaped particles with dense structure. As clearly seen in Fig. 1A and B the surface of the obtained crystals is smooth and without any visible pores. The carbonized MOF-5 exhibited a typical cubic shape with lower density in contrast to MOF-5. In MOF-5 after carbonization, the porous structure with additional cracks and cavities was observed.^38^ TEM image analysis, presented in Fig. 2A–D, shows the pore structure of the carbonized MOF-5. The cracks observed in Fig. 1D and E are also clearly observed in TEM images (Fig. 2C). They were formed as a consequence of the extraction of zinc oxide from the interior of MOF-5. The EDS analysis shows peaks attributed to oxygen, carbon and copper (Fig. 2E). Copper signals originate from the TEM grid. The absence of the peaks corresponding to the zinc proves the efficient synthesis of the pure carbon structures from MOF-5. The transmission electron microscopy images confirms the SEM observation regarding the low density and shape of the carbonized MOF-5. SEM image analysis (Fig. 1C and F) shows that MOF-5 and carbonized MOF-5 structures exhibit similar size distribution. The reported size of the MOF-5 was mostly in the range of 3.75–5 μm. During carbonization cubic structures have shrunk slightly and size of the CMOF-5 was mostly in the range of 2.75–4.5 μm.

Additionally, Raman spectroscopy confirms the presence of carbon in the sample. As shown in Fig. 2F, the Raman spectrum shows two strong peaks at around 1300 and 1600 cm^−1^ corresponding to the D and G bands, respectively. The D band is ascribed to the vibration of carbon atoms with dangling bonds in the plane with termination by disordered graphite. The G band indicates the E_2_g mode in carbon with high graphitization degree and it is related to the vibration of sp^2^-hybridized carbon atoms. The D band has much higher intensity than G band which suggests that carbonized MOF-5 have a lot of defects and it consist mostly of amorphous carbon.

**Fig. 3:**
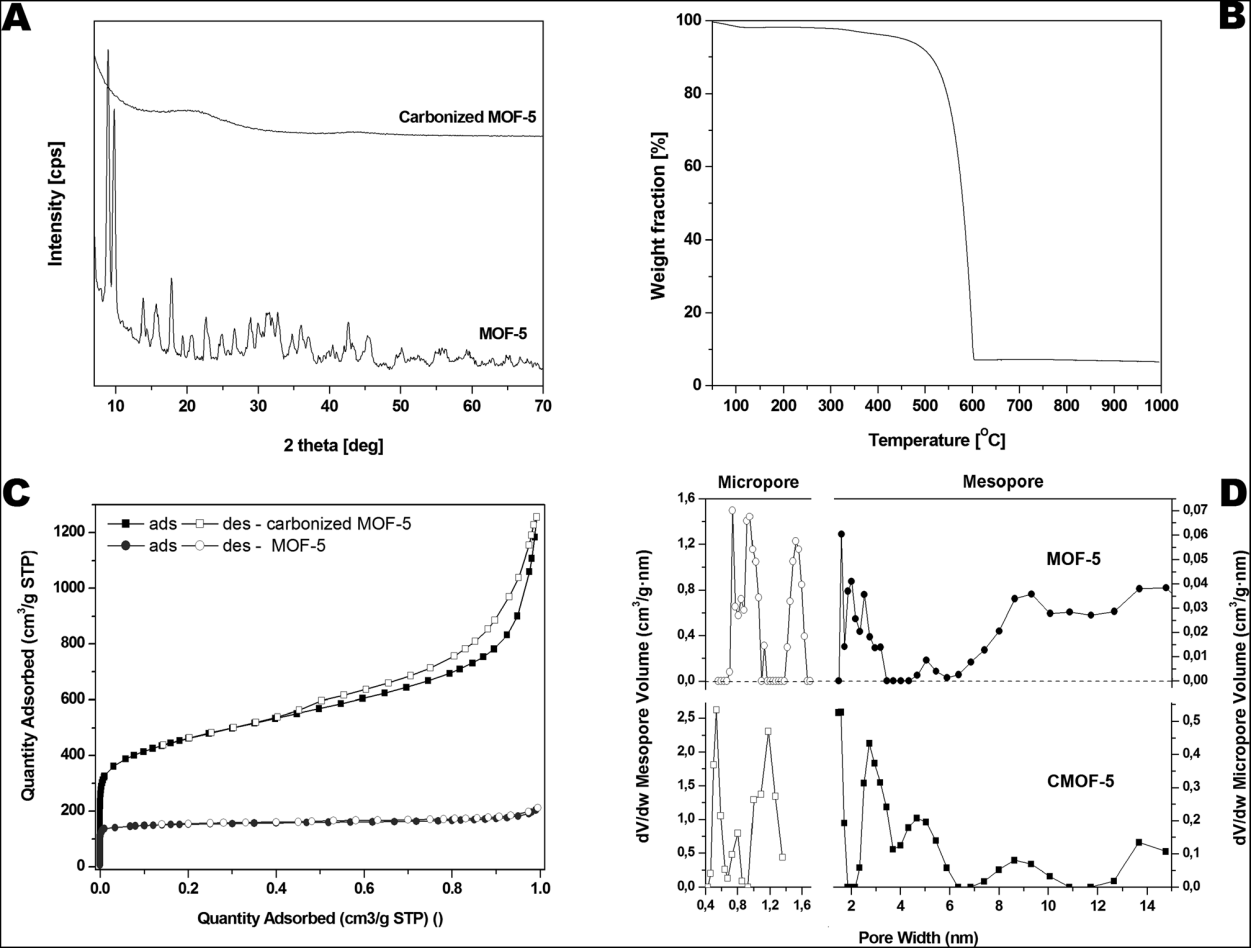
Pristine and carbonized MOF-5 XRD spectrum (A), TGA (B), nitrogen adsorption/desorption profile (C) and pore volume distribution in carbonized MOF-5 (D).

The crystal structure of MOF-5 before and after carbonization was investigated by X-ray diffraction (XRD) (Fig. 3A). All reflections in XRD pattern of MOF-5 before carbonization can be attributed to the reference standard card (CCDC – 256965). The reflections at 2*θ* angle of ∼7°, 9.8°, 13.8° and 15.6° correspond to the (002), (022), (004), and (024) planes, respectively.^59^ There are no significant peaks corresponding to zinc oxide in XRD pattern of MOF-5 after the carbonization. The carbonized sample shows broad peaks between 20°, 25° and at ∼45°, related to the disorderly oriented carbon with low crystallinity. The presented XRD data are in good accordance with morphology analysis demonstrating: (i) metal species extraction and (ii) high purity of carbon material obtained during the carbonization process.

Furthermore, thermogravimetric analysis was performed in the temperature range from room temperature to 1000 °C under inert argon gas flow and presented in the Fig. 3B. A small weight loss assigned to the removal of water and residual solvent molecules is observed in the temperature from 100 to 200 °C (up to 2 wt%). Next weight loss starts around 385 °C and ends around 600 °C. It is related to the thermal decomposition of carbonized MOF-5 and the formation of carbon dioxide. The weight loss was ∼90 wt% at 1000 °C which indicates that some residual metal species are still present in the sample.

The porosity of the pristine and carbonized MOF-5 was tested by the N_2_ adsorption–desorption experiment. The typical IV isotherms with H3 hysteresis loops are observed in the samples, which is typical of mesoporous materials (Fig. 3C). The hysteresis loop for pristine MOF-5 is not clearly seen because the porosity of this sample is considerably lower than the porosity of carbonized MOF-5. The pore size distribution curves show the coexistence of micro- and mesopores below 10 nm in both samples but the micropore and mesopore volumes are much higher for carbonized MOF-5. The calculated BET specific surface area, total pore volume and micropore volume was 1884 m^2^ g^−1^, 1.84 cm^3^ g^−1^ and 0.59 cm^3^ g^−1^, respectively. The specific surface area, total pore volume and micropore volume estimated for pristine MOF-5 were equal to 477 m^2^ g^−1^, 0.33 cm^3^ g^−1^ and 0.24 cm^3^ g^−1^, respectively. During carbonization the porous structure was built up.

Based on the above results we can conclude that during carbonization some kind of activated carbon was obtained. XRD and Raman results confirmed formation of amorphous carbon. N_2_ adsorption–desorption measurements showed that highly porous carbon was obtained during carbonization. The changes of textural properties of MOF-5 are presented at Fig. 3C and D.

## 4. Gas absorption properties

**Fig. 4:**
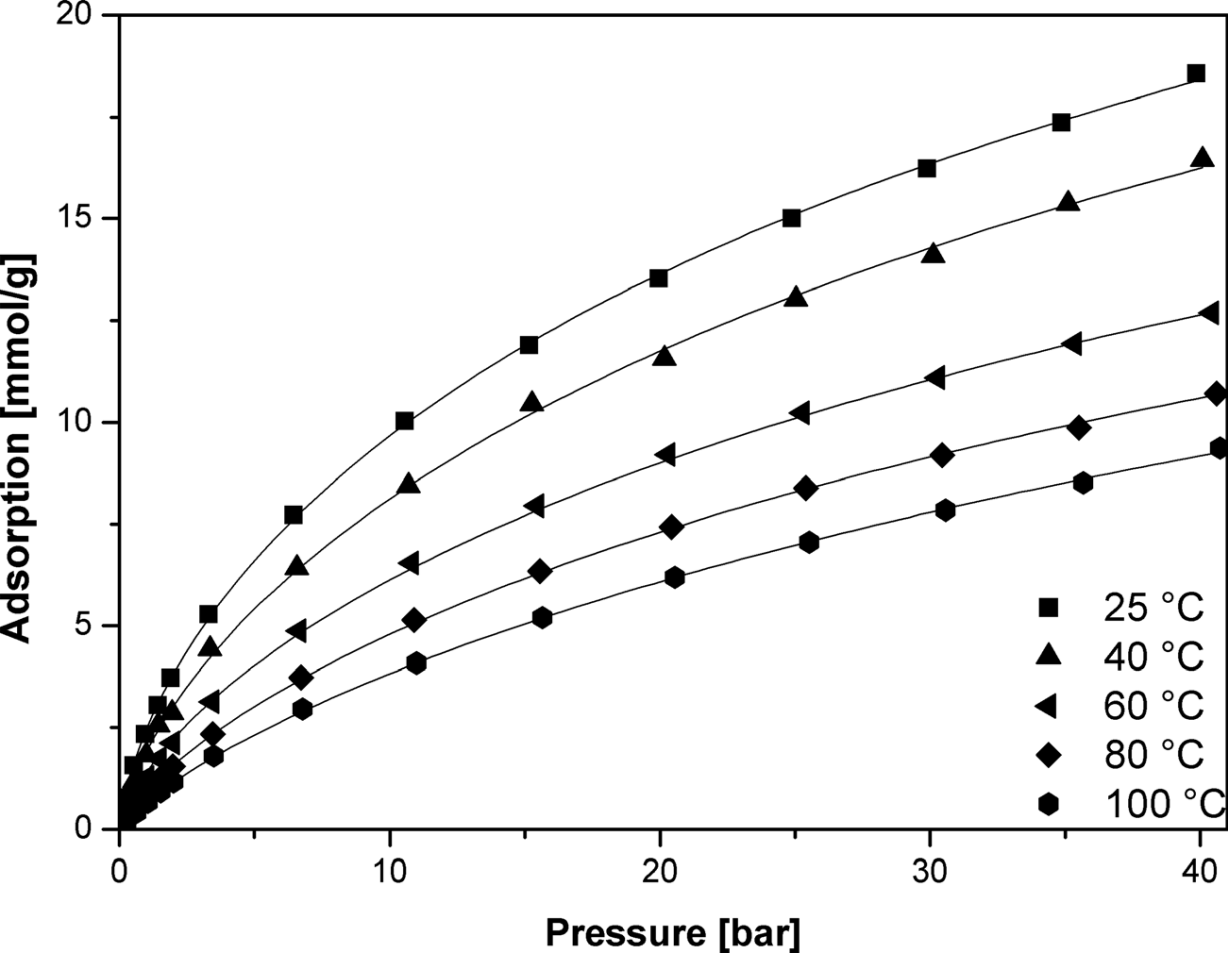
CO_2_ adsorption isotherms in carbonized MOF-5 (points – experimental data, lines – calculated using the Sips equation).

The data for adsorption of carbon dioxide in carbonized MOF-5 were collected in the temperature ranging from 298 to 373 K and pressure up to 40 bar (Fig. 4).

The carbon dioxide adsorption isotherms were fitted using Freundlich, Langmuir, Sips and Toth empirical equations. More detailed information about these models can be found elsewhere.^60,61^

The adsorption isotherm data were fitted to the isotherm models by non-linear regression method. The reduced chi-squared was applied to test the how the models fit to experimental data. The smaller the reduced chi-squared value, better fitting is assumed. Basing on it, it was found that the Sips model provided the most accurate fit to the CO_2_ adsorption data. The Sips model is also called the Langmuir–Freundlich model because it contains the elements of Langmuir and Freundlich models. Sips model is similar to Freundlich model, but it has a finite limit of the sufficiently high pressure:1
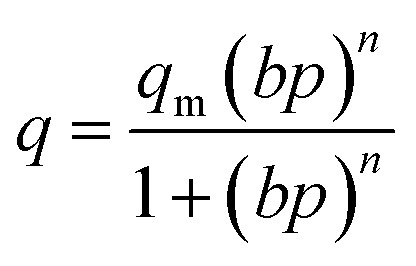
*q* [mmol g^−1^] – the amount of adsorbed CO_2_ at given temperature and equilibrium pressure *p*; *q*_m_ [mmol g^−1^] – the maximum amount of adsorbed CO_2_ at given temperature; *b* [1/bar] – parameter called the affinity constant; *n* – dimensionless parameter characterized the system heterogeneity.

Table 1 shows the Sips isotherms parameters and their errors and reduced chi-sqr and adjusted *R*-square (*R*^2^).

**Table 1:** The calculated Sips isotherms parameters

Temp. [K]	*q*_m_ [mmol g^−1^]		*b* [1/bar]		*n*		Chi-sqr	*R* ^2^
	Value	Error	Value	Error	Value	Error		
298	44.6	2.7	0.0147	0.0023	0.672	0.013	0.0097	0.9998
313	42.5	5.2	0.0124	0.0037	0.694	0.025	0.0241	0.9993
333	35.1	5.1	0.0111	0.0061	0.706	0.041	0.0324	0.9991
353	30.3	6.2	0.0110	0.0051	0.759	0.038	0.0399	0.9990
373	28.6	2.5	0.0098	0.0017	0.806	0.017	0.0025	0.9998

The parameter *b* is a measure of strongness of adsorbate and sorbent interaction. The values of parameter *b* decreased with the increase in the temperature. Therefore, the adsorbate and sorbent interaction is weaken when the temperature is elevated.

The parameter *n* is a measure of heterogeneity of the adsorbate and sorbent system. If the *n* is equal 1 the system is homogenous and the Sips equation is reduced to the Langmuir equation. The lower is *n* parameter the more heterogeneous is the system. In our study the values of *n* parameter increased along with the temperature. It is indication that the heterogeneity of our system increased at higher temperature. The changes of *b* and *n* parameters were in agreement with the multitemperature Sips equation. The temperature dependence of the affinity constant *b* is the following:^54^2
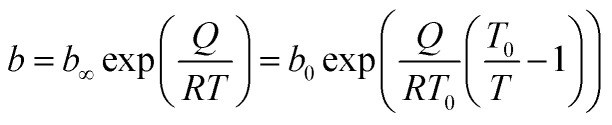
*b*_∞_ [1/bar] – adsorption affinity constant at infinite temperature; *b*_0_ [1/bar] – adsorption affinity constant at reference temperature *T*_0_; *Q* [J mol^−1^] – measure of the adsorption heat; *T*_0_ [K] – is the reference temperature that can be chosen arbitrarily; *R* [J (mol^−1^ K^−1^)] – the gas constant.

The temperature dependence of the exponent *n* is the following:^54^3
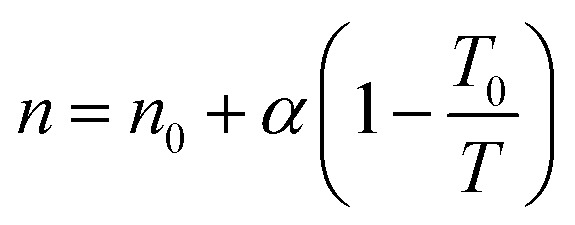
*n*_0_ – dimensionless parameter characterized the system heterogeneity at reference temperature *T*_0_; *α* – dimensionless constant parameter.

The saturation capacity *q*_m_ can be consider as a constant. However, it can also following the temperature dependence:^62^4
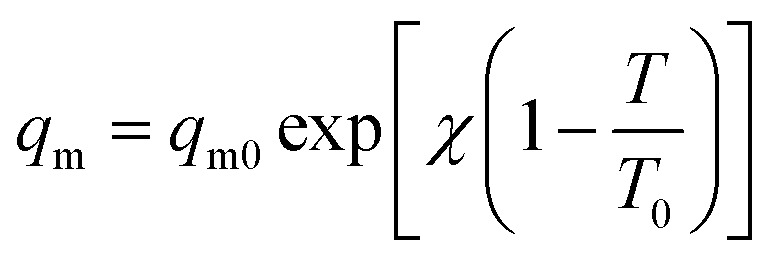
*χ* – dimensionless constant parameter.

In order to calculate the parameters in (2)–(4) equations, they were linearized and plotted:

5 ln(*q*_m_) = *f*_1_(*T*)

6 ln(*b*) = *f*_2_(1/*T*)

7 *n* = *f*_3_(1/*T*)

**Fig. 5:**
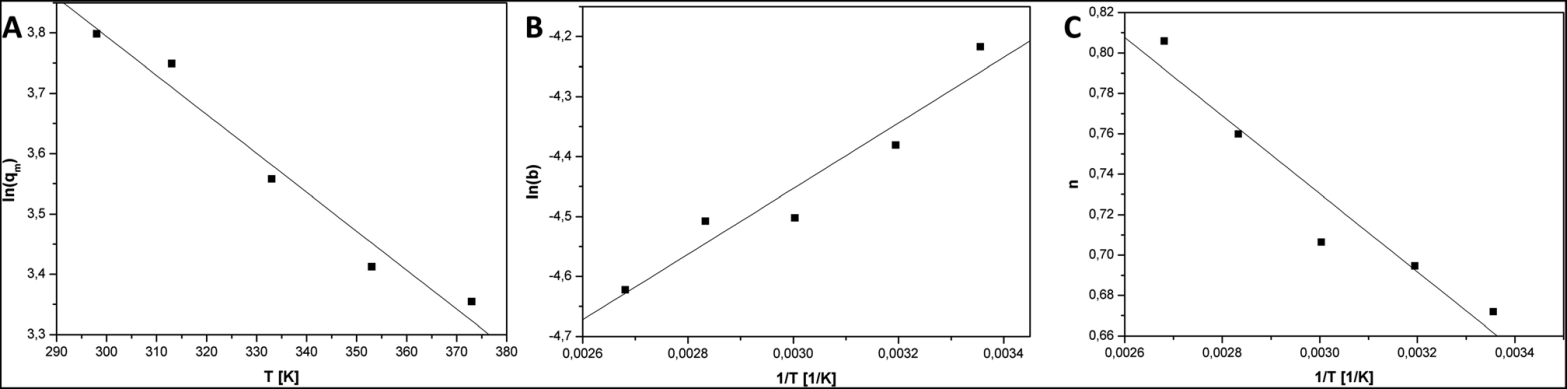
Plots of ln(*q*_m_) *vs. T* (A), ln(*b*) *vs.* 1/*T* (B) and *n vs.* 1/*T* (C).

Fig. 5 show plots of functions: ln(*q*_m_) = *f*_1_(*T*), ln(*b*) = *f*_2_(1/*T*), *n* = *f*_3_(1/*T*). Table 2 present the results of the linear fitting. The slope is denoted as *a*_1_ and the intercept as *a*_0_.

**Table 2:** The results of the linear fitting of (5)–(7) functions

Function	*a*_1_		*a*_0_		*R* ^2^
	Value	Error	Value	Error	
ln(*q*_m_) = *f*_1_(*T*)	5.73	0.22	−0.00646	0.00066	0.9597
ln(*b*) = *f*_2_(1/*T*)	−6.10	0.27	547	88	0.9027
*n* = *f*_3_(1/*T*)	1.310	0.094	−193	31	0.9034

On the basis on the values listed in Table 2 the optimal parameters for the temperature dependent Sips equations ((2)–(4)) were calculated (Table 3). *T*_0_ was the reference temperature that was equal to 298 K as the lowest temperature set in the sorption investigations of carbonized MOF-5.

**Table 3:** The optimal parameters for the temperature dependent Sips equations. Reference temperature *T*_0_ = 298 K

Parameter	Value	Error
*Q* [J mol^−1^]	4550	68
*b*_0_ [1/bar]	0.0141	0.0058
*n*_0_	0.662	0.035
*α*	0.648	0.026
*q*_m0_ [mmol g^−1^]	45.0	2.2
*χ*	1.925	0.016

As it is presented, the multitemperature Sips model (eqn (1)–(4)) provided a very successful fit of the adsorption observed in the experimental data. Therefore, the calculated Sips equation parameters can be used for isosteric heat of adsorption calculation. Isosteric heat of adsorption (*Q*_is_) is one of the basic requirements for the characterization and optimization of the adsorption process. It is defined as the heat of adsorption determined at constant surface coverage (*θ*). A surface coverage is defined as:8
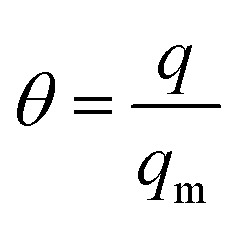


The isosteric heat of adsorption was calculated using the Clausius–Clapeyron eqn (9):9
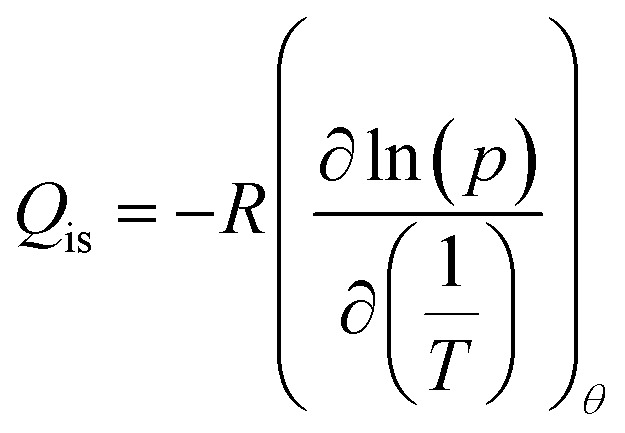


The calculated parameters of Sips equation (Table 1) were used to calculate the pressure values at different temperatures, at constant surface coverage from (10):10
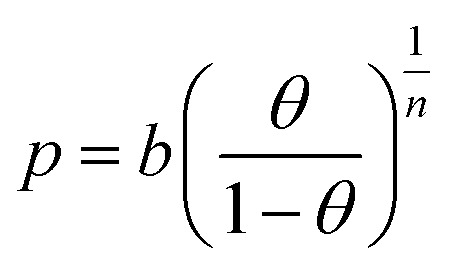


**Fig. 6:**
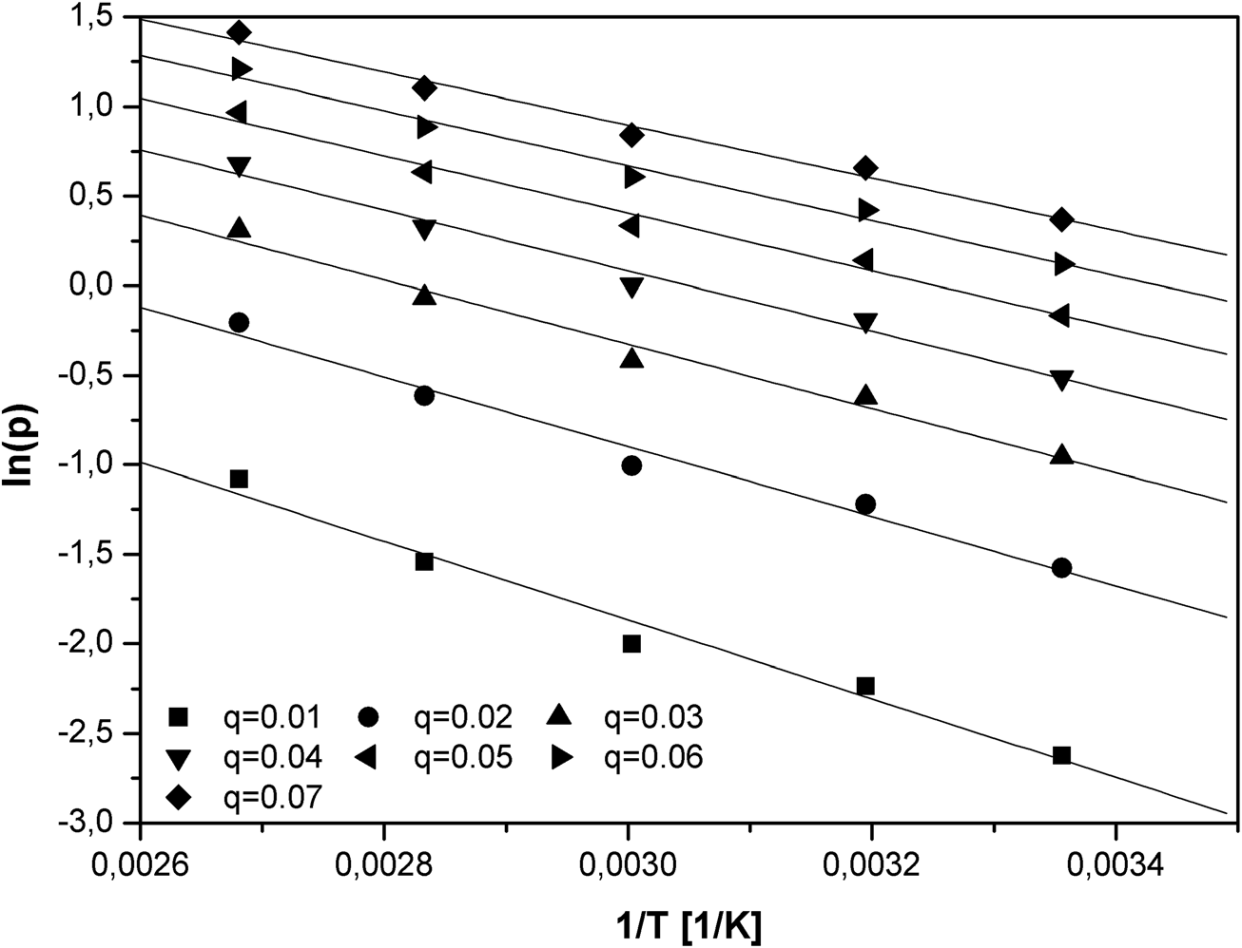
The plot of ln(*p*) *vs.* 1/*T* for constant surface coverage.

The logarithm of the equilibrium pressures (ln(*p*)) was plotted against the reciprocal temperature (1/*T*) at the constant coverage *θ* (Fig. 6). The surface coverage was varied from 0.01 to 0.07 with interval of 0.01.

The isosteric heat of adsorption was calculated utilizing the slopes (*A*) of the linear functions ln(*p*) = *f*(1/*T*):

11 *Q*_is_ = −*RA*

**Fig. 7:**
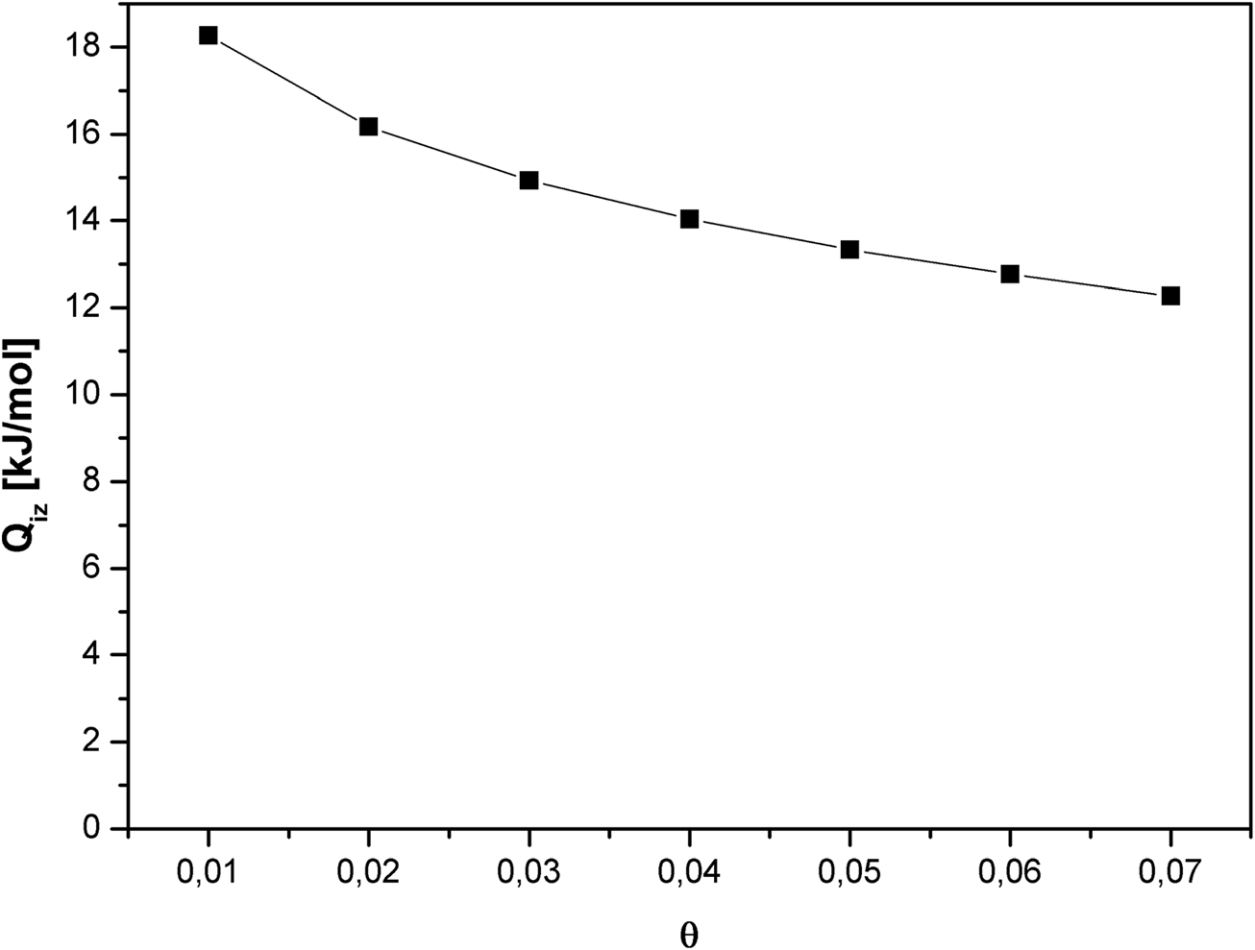
Isosteric heat of adsorption as a function of surface coverage.

The isosteric heat of adsorption for CO_2_ decreased substantially with increase in the gas surface coverage (Fig. 7). Such course of the curve indicates the negligible intermolecular interaction between CO_2_ molecules. A decrease in the isosteric heat of adsorption with the surface coverage is a result of the disappearance of favorable adsorption sites.

Carbon dioxide molecules preferred to adsorb onto the high-energy sites. Increasing the coverage caused adsorption onto the sites of low-energy which results in a slow increase in the amount of adsorbed *vs.* pressure. This was also in agreement with the slope of adsorption isotherm (Fig. 4).

Table 4 summarize the detailed review of the data, including our data (2012–2018), on carbonized metal–organic frameworks studied as material for CO_2_ absorption. Many reports describe CO_2_ absorption results measured at 273 K (0 °C). As the temperature decreases, the absorption results increase significantly. The same applies to the use of different pressure units. Using an atmosphere or millimeters of mercury raises the result in comparison to the bars. The result then looks better (conversion factor given in the title of the Table 4). However, it is certainly necessary to unify the results in order to reliably compare them. In addition, the data including measurements at room temperature and higher temperatures seem to be more suitable for discussions on the potential in industrial application of obtained materials for CO_2_ absorption. The wide range of measured pressures and the thermodynamics of the absorption process are also valuable for testing of the potential applications of the materials. Taking these factors into account, the obtained result are among the best of materials based on carbonized metal–organic frameworks structures described in the literature. Materials based on ZIF and their modifications^44–49^ were most often discussed in the literature. Ma *et al.* obtained four different samples of carbonized MOF-5 at different time.^40^ They carried out carbonization at 600, 700, 800 and 900 °C for 5 h. Additionally, their materials were doped with nitrogen by adding urea prior carbonization. Our results of CO_2_ uptake (2.43 mmol g^−1^ at 25 °C and 1 bar) are basically very similar. This was possible by increasing the carbonization temperature to 1000 °C which led to more effective zinc oxide evaporation process^38^ and favorable development of porosity. It was realized in 2 h carbonization without any additional modifications. However, doping with nitrogen and activation of carbonaceous materials (for example using KOH) is a popular method for improving their electrochemical and absorption properties. Therefore, we do not rule out further investigation of the obtained carbonized MOF-5 after its further treatment. In this work we focused primarily on the description of the precise thermodynamics of the CO_2_ absorption process and the presentation of results in a wide range of temperatures and pressures of the pristine carbonized MOF-5.

**Table 4:** Comparison of CO_2_ uptake properties between literature and our material (1 atm = 1,01 325 bar = 760 mmHg)

Sample name	Original material	CO_2_ uptake experiment parameters	Result (mmol g^−1^)	Source
BM-900	bio-MOF-1	273 K and 1 bar	4.62	39
		298 K and 1 bar	3.55	
KBM-700	bio-MOF-1	273 K and 1 bar	4.75	39
		298 K and 1 bar	3.29	
MUC600	MOF-5	0 °C and 1 bar	3.55	40
		25 °C and 1 bar	2.44	
		25 °C and 0.15 bar	0.73	
MUC900	MOF-5	0 °C and 1 bar	3.71	40
		25 °C and 1 bar	2.31	
		25 °C and 0.15 bar	0.43	
AAC-2W	MIL-100(Al)	273 K and 1 bar	6.5	41
		298 K and 1 bar	4.8	
		298 K and 0.2 bar	1.74	
C800	MIL-100(Al)	273 K and 1 atm	4.1	42
		298 K and 1 atm	2.6	
NC800	MIL-100(Al)	273 K and 1 atm	5.7	42
		273 K and 0.15 atm	2.3	
		298 K and 1 atm	3.8	
N-HPCMs-5-0.6-973	Al-based composite	273 K and 780 mmHg	2.35	43
		298 K and 780 mmHg	1.82	
C700	ZIF-8	273 K and 1 bar	3.70	44
		298 K and 1 bar	2.76	
C1000	ZIF-8	273 K and 1 bar	4.64	44
		298 K and 1 bar	3.39	
C700W	ZIF-8	273 K and 1 bar	5.51	44
		298 K and 1 bar	3.80	
NC900	ZIF-8	273 K and 1 atm	5.1	45
		298 K and 1 atm	3.9	
AC-CB700	ZIF-8	25 °C and 1 bar	2.0	46
1000	ZIF-8	25 °C and 0.15 bar	0.99	47
		25 °C and 1 bar	3.22	
		25 °C and 20 bar	10.21	
C68	ZIF-68 + FA	273 K and 1 atm	4.76	48
C69	ZIF-69 + FA		4.54	
C70	ZIF-70 + FA		5.45	
C68	ZIF-68 + FA	298 K and 1 atm	4.00	48
C69	ZIF-69 + FA		3.86	
C70	ZIF-70 + FA		4.49	
CZIF8a	ZIF-68 + FA	273 K and 1 atm	4.04	49
CZIF68a	ZIF-69 + FA		4.49	
CZIF69a	ZIF-70 + FA		4.76	
Pristine MOF	MOF-5	25 °C and 0.15 bar	0.26	This work
		25 °C and 1 bar	1.30	
**Carbonized MOF**	**CMOF-5**	25 °C and 0.15 bar	**0.57**	**This work**
		25 °C and 1 bar	**2.43**	
		25 °C and 10 bar	**9.73**	
		25 °C and 20 bar	**13.55**	
		25 °C and 40 bar	**18.56**	
		40 °C and 0.15 bar	**0.39**	
		40 °C and 1 bar	**1.95**	
		40 °C and 10 bar	**8.09**	
		40 °C and 20 bar	**11.76**	
		40 °C and 40 bar	**16.27**	
		100 °C and 0.15 bar	**0.12**	
		100 °C and 1 bar	**0.66**	
		100 °C and 10 bar	**3.86**	
		100 °C and 20 bar	**6.15**	
		100 °C and 40 bar	**9.18**	

**Fig. 8:**
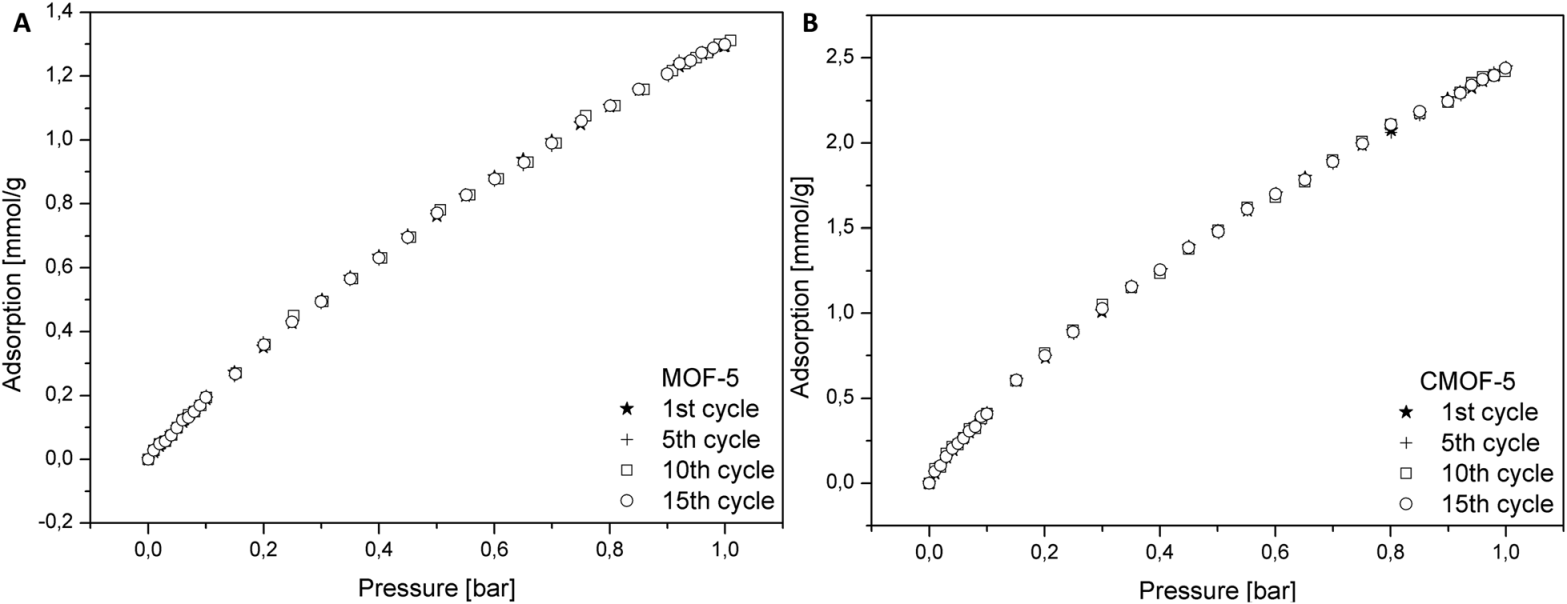
Cycling stability of pure MOF-5 (A) and carbonized MOF-5 (B).

In order to determine the regeneration performance of the obtained samples, pure and carbonized MOF-5 were subjected to a fifteen cycles of adsorption and desorption. The data for adsorption of carbon dioxide in pristine and carbonized MOF-5 were collected in the temperature of 25 °C and pressure up to 1 bar. As presented in the Fig. 8, the performance of adsorption after fifteen cycles does not change for both materials. It also turns out that the material obtained by carbonization allows adsorption to be 1.87 times higher (at 1 bar) than in the case of pure MOF-5. This analysis indicated that the adsorption of CO_2_ did not influence the efficiency of MOF-5 and carbon derived MOF-5 structures.

## 5. Conclusions

The above results revealed that the obtained carbonized MOF-5 can be successfully used in CO_2_ absorption. The CO_2_ uptake was 2.43 mmol g^−1^ at 25 °C and 1 bar. The result is similar to the best described earlier in the literature. However, in our case the result was obtained without additional modifications of the material (such as nitrogen doping or KOH activation) but only by increasing the carbonization temperature to 1000 °C.

The total pore volume after carbonization increased five times, specific surface area increased four times and micropore volume two and half times. This is the reason that CO_2_ uptake at carbonized MOF-5 was nearly two times higher than at pristine MOF-5.

Additionally, the absorption in a wide range of temperatures (25, 40, 60, 80 and 100 °C) and pressures (in range of 0–40 bar) was investigated. These temperature range is less popular because it can result in lower values. However, it is at the same time this where the potential applications of technology in the industry have place. The points on the absorption chart were experimental data, and the isotherms were calculated using the Sips equation. In this work, thermodynamics of the absorption process was also precisely described. This gives a broader perspective on the potential use of material in industrial practice.

## Conflicts of interest

There are no conflicts to declare.

## Supplementary Material

## References

[cit1] Schlapbach L., Züttel A. (2001). Nature.

[cit2] Rodhe H. (1990). Science.

[cit3] Wietkamp J. (1995). Int. J. Hydrogen Energy.

[cit4] Dong J., Wang X., Xu H., Zhao Q., Li J. (2007). Int. J. Hydrogen Energy.

[cit5] Wang L., Yang R. T. (2010). Ind. Eng. Chem. Res..

[cit6] Antoniou M. K., Diamanti E. K., Enotiadis A., Policicchio A., Dimos K., Ciuchi F., Maccallini E., Gournis D., Agostino R. G. (2014). Microporous Mesoporous Mater..

[cit7] Saha D., Bao Z., Jia F., Deng S. (2010). Environ. Sci. Technol..

[cit8] Su F., Lu C. (2012). Energy Environ. Sci..

[cit9] Panella B., Hirscher M., Roth S. (2005). Carbon.

[cit10] Morris R. E., Wheatley P. S. (2008). Angew. Chem., Int. Ed..

[cit11] Ströbel R., Garche J., Moseley P. T., Jörissen L., Wolf G. (2006). J. Power Sources.

[cit12] Wang H., Gao Q., Hu J. (2009). J. Am. Chem. Soc..

[cit13] Xia Y., Yang Z., Zhu Y. (2013). J. Mater. Chem. A.

[cit14] Lozano-Castelló D., Alcañiz-Monge J., de la Casa-Lillo M., Cazorla-Amorós D., Linares-Solano A. (2002). Fuel.

[cit15] Furukawa H., Yaghi O. M. (2009). J. Am. Chem. Soc..

[cit16] Alezi D., Belmabkhout Y., Suyetin M., Bhatt P. M., Weseliński Ł. J., Solovyeva V., Adil K., Spanopoulos I., Trikalitis P. N., Emwas A.-H., Eddaoudi M. (2015). J. Am. Chem. Soc..

[cit17] Panella B., Hirscher M. (2005). Adv. Mater..

[cit18] Panella B., Hirscher M., Pütter H., Müller U. (2006). Adv. Funct. Mater..

[cit19] Rosi N. L. (2003). Science.

[cit20] Li J., Cheng S., Zhao Q., Long P., Dong J. (2009). Int. J. Hydrogen Energy.

[cit21] Yang J., Grzech A., Mulder F. M., Dingemans T. J. (2011). Chem. Commun..

[cit22] Juan-Juan J., Marco-Lozar J. P., Suárez-García F., Cazorla-Amorós D., Linares-Solano A. (2010). Carbon.

[cit23] Li Y., Yang R. T. (2007). Langmuir.

[cit24] Furukawa H., Miller M. A., Yaghi O. M. (2007). J. Mater. Chem..

[cit25] Saha D., Deng S. (2010). Tsinghua Sci. Technol..

[cit26] Wu H., Zhou W., Yildirim T. (2007). J. Am. Chem. Soc..

[cit27] Hu Y., Liu Z., Xu J., Huang Y., Song Y. (2013). J. Am. Chem. Soc..

[cit28] Babarao R., Hu Z., Jiang J., Chempath S., Sandler S. I. (2007). Langmuir.

[cit29] Stergiannakos T., Klontzas E., Tylianakis E., Froudakis G. E. (2015). J. Phys. Chem. C.

[cit30] Lin K.-S., Adhikari A. K., Ku C.-N., Chiang C.-L., Kuo H. (2012). Int. J. Hydrogen Energy.

[cit31] Teo H. W. B., Chakraborty A., Kayal S. (2017). Appl. Therm. Eng..

[cit32] Latroche M., Surblé S., Serre C., Mellot-Draznieks C., Llewellyn P. L., Lee J.-H., Chang J.-S., Jhung S. H., Férey G. (2006). Angew. Chem., Int. Ed..

[cit33] Blăniţă G., Streza M., Lazăr M. D., Lupu D. (2017). Int. J. Hydrogen Energy.

[cit34] Kayal S., Sun B., Chakraborty A. (2015). Energy.

[cit35] Ren J., Langmi H. W., North B. C., Mathe M., Bessarabov D. (2014). Int. J. Hydrogen Energy.

[cit36] Abid H. R., Tian H., Ang H.-M., Tade M. O., Buckley C. E., Wang S. (2012). Chem. Eng. J..

[cit37] Hu M., Reboul J., Furukawa S., Torad N. L., Ji Q., Srinivasu P., Ariga K., Kitagawa S., Yamauchi Y. (2012). J. Am. Chem. Soc..

[cit38] Cendrowski K., Skumial P., Spera P., Mijowska E. (2016). Mater. Des..

[cit39] Pan Y., Zhao Y., Mu S., Wang Y., Jiang C., Liu Q., Fang Q., Xue M., Qiu S. (2017). J. Mater. Chem. A.

[cit40] Ma X., Li L., Chen R., Wang C., Li H., Wang S. (2018). Appl. Surf. Sci..

[cit41] Wang J., Yang J., Krishna R., Yang T., Deng S. (2016). J. Mater. Chem. A.

[cit42] Aijaz A., Akita T., Yang H., Xu Q. (2014). Chem. Commun..

[cit43] Liu R. L., Ji W.-J., He T., Zhang Z. Q., Zhang J., Dang F. Q. (2014). Carbon.

[cit44] Bai F., Xia Y., Chen B., Su H., Zhu Y. (2014). Carbon.

[cit45] Aijaz A., Fujiwara N., Xu Q. (2014). J. Am. Chem. Soc..

[cit46] Almasoudi A., Mokaya R. (2014). J. Mater. Chem. A.

[cit47] Gadipelli S., Guo Z. X. (2015). ChemSusChem.

[cit48] Pachfule P., Biswal B. P., Banerjee R. (2012). Chem.–Eur. J..

[cit49] Wang Q., Xia W., Guo W., An L., Xia D., Zou R. (2013). Chem.–Asian J..

[cit50] Cendrowski K., Zenderowska A., Biegańska A., Mijowska E. (2017). J. Chem. Soc., Dalton Trans..

[cit51] Ming Y., Purewal J., Yang J., Xu C., Soltis R., Warner J., Veenstra M., Gaab M., Müller U., Siegel D. J. (2015). Langmuir.

[cit52] Tang J., Yamauchi Y. (2016). Nat. Chem..

[cit53] Hu M., Reboul J., Furukawa S., Torad N. L., Ji Q., Srinivasu P., Ariga K., Kitagawa S., Yamauchi Y. (2012). J. Am. Chem. Soc..

[cit54] Cendrowski K., Kukułka W., Kedzierski T., Zhang S., Mijowska E. (2018). Nanomaterials.

[cit55] Yang S. J., Kim T., Im J. H., Kim Y. S., Lee K., Jung H., Park C. R. (2012). Chem. Mater..

[cit56] Liu B., Shioyama H., Akita T., Xu Q. (2008). J. Am. Chem. Soc..

[cit57] Kimitsuka Y., Hosono E., Ueno S., Zhou H., Fujihara S. (2013). Inorg. Chem..

[cit58] Liu B., Shioyama H., Jiang H., Zhang X., Xu Q. (2010). Carbon.

[cit59] Huang L., Wang H., Chen J., Wang Z., Sun J., Zhao D., Yan Y. (2003). Microporous Mesoporous Mater..

[cit60] Sreńscek-Nazzal J., Narkiewicz U., Morawski A. W., Wróbel R. J., Michalkiewicz B. (2015). J. Chem. Eng. Data.

[cit61] Foo K. Y., Hameed B. H. (2010). Chem. Eng. J..

[cit62] Do D. D. , Adsorption Analysis: Equilibria and Thermodynamics, Imperial College Press, 1998

