# Correction: MOF-5 derived carbon as material for CO_2_ adsorption

**DOI:** 10.1039/c9ra90077b

**Published:** 2019-10-24

**Authors:** Wojciech Kukulka, Krzysztof Cendrowski, Beata Michalkiewicz, Ewa Mijowska

**Affiliations:** Nanomaterials Physicochemistry Department, West Pomeranian University of Technology, Szczecin Piastów Av. 45 Szczecin 70-311 Poland wojciech_kukulka@zut.edu.pl; Institute of Chemical and Environment Engineering, West Pomeranian University of Technology, Szczecin Pulaskiego St. 10 Szczecin 70-322 Poland

## Abstract

Correction for ‘MOF-5 derived carbon as material for CO_2_ absorption’ by Wojciech Kukulka *et al.*, *RSC Adv.*, 2019, **9**, 18527–18537.

The authors regret that the title shown in the original article and several sentences were incorrect due to the use of the word “absorption” in place of “adsorption”. The correct title is as shown above and all instances of “absorption” in the text should be “adsorption”. The Royal Society of Chemistry apologises for these errors and any consequent inconvenience to authors and readers.

## Supplementary Material

